# Living arrangements, chronic diseases, and prescription drug expenditures among Korean elderly: vulnerability to potential medication underuse

**DOI:** 10.1186/1471-2458-14-1284

**Published:** 2014-12-16

**Authors:** Eun-Ja Park, Hyun Soon Sohn, Eui-Kyung Lee, Jin-Won Kwon

**Affiliations:** Korea Institute for Health and Social Affairs, Seoul, South Korea; Graduate School of Clinical Pharmacy, CHA University, Kyeonggi-do, South Korea; College of Pharmacy, Sungkyunkwan University, Kyeonggi-do, South Korea; College of Pharmacy and Research Institute of Pharmaceutical Sciences, Kyungpook National University, 80 Daehak-ro, Buk-gu, Daegu, 702-701 South Korea

## Abstract

**Background:**

Insufficient social security combined with family structure changes has resulted in a poverty of the elderly. The objective of this study was to examine an association of living arrangements of the elderly with chronic disease prevalence and prescription drug use.

**Methods:**

2008 Korea Health Panel Survey (KHPS) data were used in this study. Information on living arrangements, socio-demographics, health behaviors, chronic disease prevalence and healthcare expenditures including out-of-pocket (OOP) prescription drug expenditures for elderly aged 65 or older were collected from self-reported diaries and receipts. OOP prescription drug expenditure as a total cost that subject paid to a pharmacy for prescription drugs was examined. Logistic regression was used to identify differences in major chronic disease prevalence by living arrangements. The association of living arrangements with prescription drug use was analyzed using generalized linear model with a log link and a gamma variance distribution.

**Results:**

Proportions of elderly living alone, elderly living with a spouse only, and elderly living with adults aged 20–64 were 14.5%, 48.3%, and 37.2%, respectively. Elderly living alone showed 2.43 odds ratio (OR) (95% confidence interval (CI) = 1.66-3.56) for having major chronic diseases prevalence compared to elderly living with adults. Despite a higher major chronic disease prevalence, elderly living alone showed lower OOP prescription drug expenditures (Cost Ratio = 0.80, 95% CI = 0.67-0.97) after adjusting for the number of major chronic diseases. Total OOP prescription drug expenditures were significantly lower in patients with a low income level versus high income level.

**Conclusions:**

Even though elderly living alone had a higher risk of chronic disease, they spent less on OOP prescription drug expenditures. Optimal drug use is important for elderly with chronic diseases to achieve good health outcomes and quality of life. Public health policies should be supplemented to optimize medical treatment for vulnerable elderly living alone.

## Background

Several Asian countries including South Korea are confronting a rapidly aging population. The proportion of elderly aged 65 and over among the total Korean population increased from 3.8% in 1980 to 11.0% in 2010 and is estimated to be 24% in 2030 [1]. As Korean baby boomers become parts of the elderly population, this population will continue to increase. With the population aging, Asian countries have experienced a rapid industrialization and have changed to a westernized life style [2]. These changes have led to elderly people living alone or only with a spouse instead of living with their children. The proportions of elderly living alone (13.6% and 19.6%) and with a spouse (26.8% and 47.0%) among the entire elderly population in South Korea increased by 44% and 75%, respectively, from 2004 to 2011 [3]. Several studies have reported that living arrangements like living alone led to a poor self-rated health and high levels of disability and depression in elderly group vulnerable to chronic diseases [4–8]. But, it has not been studied how living arrangements relate to chronic diseases in the elderly.

Growing drug use in the elderly has led to drug expenditure increases, and recent statistics in 2005 reported a 21.3% increase in total drug expenditures by the elderly compared to that in 2001 [9–11]. This figure itself has engaged social concerns, but living arrangements for the elderly as a factor possibly influencing healthcare utilization has received less attention. Elderly, especially those living alone, have a higher risk of social isolation [12], but many Asian countries do not have a sufficient social security system such as old-age pension. An insufficient social security with living arrangement changes has resulted in a poverty of the elderly.

The South Korean government is trying to strengthen a welfare for the elderly, but South Korea still has the highest elderly poverty rate among OECD countries (45%) and elderly living alone were poorer than elderly living with a spouse or other adult [13]. Low income and social isolation in elderly living alone or with a spouse likely influence medication uses. There is no doubt that patient charges can reduce the use of prescription drugs [14], but the user charge burden as out-of-pocket (OOP) money among the elderly has mostly been investigated in Western countries, not Asian countries [15]. In South Korea, the prescription drugs listed in the national formulary are basically covered by National Health Insurance (NHI) with a 30% co-insurance payment. But for the elderly, for a prescription drug costing less than US$9, the user charge is fixed at US$1. However, in 2010, only 2.4% of all prescription cost were less than US$9 [16].

The objective of this study was to examine the association of living arrangements of the elderly to chronic disease prevalence and prescription drug use. Specifically, we explored whether elderly living alone were vulnerable to potential medication underuse compared to elderly living with a spouse or other adults.

## Methods

### Data and study population

2008 Korea Health Panel Survey (KHPS) data were used in this study. KHPS, a panel study conducted by Korea Institute for Health and Social Affairs (KIHASA) that has examined health care utilization and expenditures of national populations in South Korea since 2008, used a housing unit sampling frame based on the Korean Census and selected household samples using a 2-stage cluster sampling method stratified by residence area. Information on socio-demographic characteristics, health status, health care utilization and health care expenditures for all household members was collected using questionnaires and diaries. This study was exempted from institutional review board (IRB) approval based on the regulation that any researches using KIHASA-generating database funded by government and open to the general public through KIHASA website were exempted from the IRB review process.

A total of 21,787 subjects were enrolled in 2008 KHPS. Among them, 2,881 were elderly aged 65 and older. We excluded subjects who were Medical Aid beneficiaries under a different health plan without a user charge burden for prescription drugs, those for whom we had no basic information on education, income, smoking status, or medication use, and those who were living with only children aged 19 or less or with another elderly person besides a spouse. Finally, 2,342 elderly subjects were included in the analysis.

### Measures

#### Living arrangements

Living arrangement was investigated from the question “What is your family structure?” We classified family structure of elderly enrolled in this study into three groups: living alone, living only with spouse, and living with adults aged 20–64.

#### Socio-demographic characteristics

Subjects were classified as younger elderly (65 to 74 years old) and older elderly (more than 75 years old) and by gender. Educational attainment, the highest level of education completed, was classified into three groups: elementary school or less, middle school, and high school or more. Employment was classified as not employed or employed. Household equivalent income was defined as the total household income divided by the square root of the number of household members, and was classified into four groups using quartiles. Household income included salaries, self-employment income, public or private transfer income, interest, and so on.

#### Health behaviors

Personal smoking status was categorized into 3 groups: non-smoker (smoked < 100 cigarettes until now), current smoker (smoked ≥ 100 cigarettes until now and still smoking), or ex-smoker (smoked ≥ 100 cigarettes until now but now quitted). Exercise behavior was categorized as yes (participation in physical activity lasting at least 20 minutes per day three times per week or more) or no. Body Mass Index (BMI) was calculated by dividing weight in kilograms by the square of height in meters, using self-reported weight and height figures. Obesity was defined by BMI classified into three groups: normal (BMI < 23), overweight (23 to <25), and obese (≥25).

#### Chronic disease prevalence

Subjects were asked to report chronic diseases which were physician-diagnosed in order to increase reliability of self-reported disease prevalence. Their reported chronic diseases were classified using 298 disease categories defined the Korean Standard Classification of Diseases and Causes of Death (KSCD) based on the tenth version of International Statistical Classification of Diseases and Related Health Problems (ICD-10). The most prevalent 10 chronic diseases in elderly groups including hypertension, arthritis, diabetes, dorsopathies and ankylosing spondylitis, disorders of bone density and structure including osteoporosis, gastritis and duodenitis, cataract and disorders of lens, disorders of lipoprotein metabolism, soft tissue disorders including neuralgia and Intervertebral disc disorder, were considered as major chronic diseases in this study. We classified the number of major chronic diseases per subject into three groups: none, one, and two or more.

#### Healthcare utilization and medical expenditure

Healthcare utilization information such as hospitalization, physician visits and prescription drug fills, and out-of-pocket (OOP) costs paid by subjects during 2008 were collected from receipts that each subject kept and a diary for self-reporting each event. OOP medical expenditures including overall subject burden for hospital stays, outpatient services, emergency room visits, and outpatient prescriptions that were not covered by the national health insurance were measured. More specific OOP prescription drug expenditures as the total cost that subject paid to pharmacy for prescription drug fill were examined for medication use focusing analysis.

### Statistical analysis

Descriptive analysis was conducted by living arrangements of the elderly subjects. Binary logistic regression was conducted to identify difference in chronic disease prevalence by living arrangements. Socio-demographic characteristics and health behaviors such as smoking, exercise and obesity were used as explanatory variables. The associations of living arrangements with OOP medical expenditures and prescription drug expenditures were examined using the generalized linear model (GLM) with a log link and a gamma variance distribution. Statistical analyses were performed using the SAS software version 9.1 (SAS Institute Inc., Cary, NC, USA) and STATA software version 10.0 (Stata Corp, College Station, TX, USA). The statistical significance level was set at p < 0.05, two-sided.

## Results

**Figure 1:**
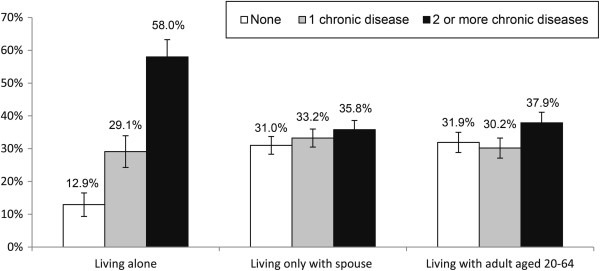
**Major chronic disease prevalence by living arrangements in the elderly.**

Among 2,342 subjects, 340(14.5%) lived alone, 1,131 (48.3%) lived with a spouse only, and 871(37.2%) lived with adults aged 20–64. Among the subjects living alone, 111 (32.7%) were very old (aged 75 or older) and most (88.5%) were women. The elderly living alone were less educated, had a lower income level, and were more obese than elderly living with someone (Table 1). Nearly 70% of elderly persons had more than one major chronic disease, and frequently prevalent chronic diseases were hypertension (44.9%), arthritis (23.5%) and diabetes (15.9%). Elderly living alone showed a higher comorbidity rate of two or more major chronic diseases than elderly living with a spouse or other adult (58.0% vs. 35.8% or 37.9%) (Figure 1).

**Table 1:** **Subject characteristics and living arrangements**

Characteristics	Total (n, %)	Living arrangement (n, %)		
		Living alone	Living only with spouse	Living with adult aged 20-64
All	2,342 (100.0)	340 (100.0)	1,131 (100.0)	871 (100.0)
Age				
65-74	1759 (75.1)	229 (67.3)	920 (81.3)	610 (70.0)
75+	583 (24.9)	111 (32.7)	211 (18.7)	261 (30.0)
Gender				
Women	1306 (55.8)	301 (88.5)	477 (42.2)	528 (60.6)
Men	1036 (44.2)	39 (11.5)	654 (57.8)	343 (39.4)
Education				
Elementary school or less	1535 (65.5)	284 (83.5)	664 (58.7)	587 (67.4)
Middle school	297 (12.7)	21 (6.2)	162 (14.3)	114 (13.1)
High school or more	510 (21.8)	35 (10.3)	305 (27.0)	170 (19.5)
Employment				
Not employed	1400 (59.8)	213 (62.7)	579 (51.2)	608 (69.8)
Employed	942 (40.2)	127 (37.3)	552 (48.8)	263 (30.2)
Household equivalent income^†^				
High	599 (25.6)	22 (6.4)	181 (16.0)	396 (45.4)
Middle high	595 (25.4)	52 (15.3)	276 (24.4)	267 (30.7)
Middle low	567 (24.2)	109 (32.1)	323 (28.6)	135 (15.5)
Low	581 (24.8)	157 (46.2)	351 (31.0)	73 (8.4)
Smoking				
Non-smoker	1374 (58.6)	275 (80.9)	579 (51.2)	520 (59.7)
Ex-smoker	613 (26.2)	35 (10.3)	363 (32.1)	215 (24.7)
Current-smoker	355 (15.2)	30 (8.8)	189 (16.7)	136 (15.6)
Exercise				
No	2114 (90.3)	322 (94.7)	989 (87.4)	803 (92.2)
Yes	228 (9.7)	18 (5.3)	142 (12.6)	68 (7.8)
BMI (kg/m^2^)				
Normal (<23)	1209 (51.6)	164 (48.2)	586 (51.8)	459 (52.7)
Overweight (23 to <25)	592 (25.3)	73 (21.5)	314 (27.8)	205 (23.5)
Obese (≥25)	541 (23.1)	103 (30.3)	231 (20.4)	207 (23.8)
No of major chronic diseases				
None	673 (28.7)	44 (12.9)	351 (31.0)	278 (31.9)
1	738 (31.5)	99 (29.1)	376 (33.2)	263 (30.2)
2 or more	931 (39.8)	197 (58.0)	404 (35.8)	330 (37.9)
Major chronic diseases				
Hypertension	1,051 (44.9)	176 (51.8)	486 (43.0)	389 (44.7)
Arthritis	550 (23.5)	130 (38.2)	222 (19.6)	198 (22.7)
Diabetes	373 (15.9)	57 (16.8)	181 (16.0)	135 (15.5)
Dorsopathies and ankylosing spondylitis	237 (10.1)	54 (15.9)	103 (9.1)	80 (9.2)
Disorders of bone density and structure including osteoporosis	234 (10.0)	57 (16.8)	96 (8.5)	81 (9.3)
Gastritis and duodenitis	192 (8.2)	31 (9.1)	103 (9.1)	58 (6.7)
Cataract, disorders of lens	170 (7.3)	36 (10.6)	75 (6.6)	59 (6.8)
Disorders of lipoprotein metabolism	154 (6.6)	36 (10.6)	69 (6.1)	49 (5.6)
Soft tissue disorders including neuralgia	118 (5.0)	22 (6.5)	50 (4.4)	46 (5.3)
Intervertebral disc disorder	113 (4.8)	29 (8.5)	57 (5.0)	27 (3.1)

^†^High (≥ US$13,166); Middle high (US$7,760 to <13,166); Middle low (US$4,690 to <7,760); Low (<US$4,690).

Table 2 presents the logistic regression results for chronic disease prevalence by subject characteristics. The likelihoods of having major chronic diseases were 2.43-fold higher (95% CI = 1.66-3.56) respectively in elderly living alone than in elderly living with an adult aged 20–64. Significantly lower odds ratios of major chronic disease prevalence were observed in men versus women. Major chronic diseases were more prevalent in overweight or obese elderly than in normal.

**Table 2:** **Factors contributing to chronic disease prevalence in elderly: logistic regression**

	1 major chronic diseases or more, odds Ratio (95% confidence interval)
Living arrangement	
Living with adult aged 20-64	1.00
Living only with spouse	1.29 (1.03-1.61)*
Living alone	2.43 (1.66-3.56)*
Age	
65-74	1.00
75+	1.24 (0.98-1.57)
Gender	
Women	1.00
Men	0.39 (0.29-0.53)*
Education	
Elementary school or less	1.00
Middle school	0.89 (0.66-1.19)
High school or more	1.06 (0.81-1.37)
Employment	
Not employed	1.00
Employed	0.91 (0.74-1.12)
Household equivalent income^†^	
High	1.00
Middle high	0.98 (0.75-1.28)
Middle low	1.02 (0.76-1.36)
Low	0.98 (0.72-1.33)
Smoking	
Non-smoker	1.00
Ex-smoker	1.16 (0.85-1.57)
Current smoker	0.83 (0.60-1.15)
Exercise	
No	1.00
Yes	0.93 (0.68-1.26)
BMI (kg/m^2^)	
Normal (<23)	1.00
Overweight (23 to <25)	1.98 (1.57-2.51)*
Obese (≥25)	2.54 (1.95-3.30)*

^†^High (≥ US$13,166); Middle high (US$7,760 to <13,166); Middle low (US$4,690 to <7,760); Low (<US$4,690).

*P-value < 0.05.

Total OOP medical expenditure (Cost ratio = 0.65, 95% CI = 0.48-0.89) and OOP prescription drug expenditure (Cost ratio = 0.80, 95% CI = 0.67-0.97) were significantly less for elderly living alone than for elderly living with another adult aged 20–64, after adjusting for age, gender and number of major chronic diseases (Table 3, Model 2). However, the differences were not significant after adjusting for education, employment, and household income status (Table 3, Model 3). Total OOP prescription drug expenditure was significantly lower at low income level compared to high income level.

**Table 3:** **Factors contributing to out-of-pocket (OOP) medical expenditure and prescription drug expenditure in elderly**

	OOP medical expenditure cost ratio (95% confidence interval)			OOP prescription drug expenditure cost ratio (95% confidence interval)		
	Model 1	Model 2	Model 3	Model 1	Model 2	Model 3
Living arrangement						
Living with adult aged 20-64	1.00	1.00	1.00	1.00	1.00	1.00
Living only with spouse	0.86	0.82	1.05	0.97	0.94	1.10
	(0.72-1.04)	(0.66-1.01)	(0.84-1.32)	(0.87-1.08)	(0.82-1.07)	(0.96-1.26)
Living alone	0.76	0.65	0.85	0.96	0.80	0.98
	(0.58-0.98)*	(0.48-0.89)*	(0.62-1.16)	(0.83-1.13)	(0.67-0.97)*	(0.81-1.18)
Age						
65-74	1.00	1.00	1.00	1.00	1.00	1.00
75+	1.05	1.08	1.05	1.10	1.16	1.11
	(0.87-1.27)	(0.87-1.35)	(0.85-1.31)	(0.98-1.24)	(1.02-1.33)*	(0.97-1.27)
Gender						
Women	1.00	1.00	1.00	1.00	1.00	1.00
Men	1.06	1.26	1.28	0.99	1.41	1.42
	(0.89-1.26)	(1.03-1.55)*	(1.03-1.60)*	(0.89-1.10)	(1.24-1.61)*	(1.24-1.63)*
No. of major chronic diseases						
0		1.00	1.00		1.00	1.00
1		1.63	1.67		2.11	2.17
		(1.27-2.09)*	(1.32-2.12)*		(1.81-2.46)*	(1.88-2.52)*
2+		2.31	2.39		4.18	4.33
		(1.81-2.96)*	(1.89-3.03)*		(3.58-4.87)*	(3.73-5.03)*
Education						
Elementary school or less			1.00			1.00
Middle school			0.82			1.06
			(0.61-1.09)			(0.88-1.26)
High school or more			1.04			1.12
			(0.80- 1.35)			(0.95-1.31)
Employment						
Not employed			1.00			1.00
Employed			0.76			0.68
			(0.62-0.93)*			(0.60-0.77)*
Household equivalent income^†^						
High			1.00			1.00
Middle high			0.77			1.00
			(0.59-1.00)			(0.85-1.18)
Middle low			0.71			0.84
			(0.53-0.95)*			(0.70-1.00)
Low			0.54			0.71
			(0.40-0.72)*			(0.59-0.85)*

^†^High (≥ US$13,166); Middle high (US$7,760 to <13,166); Middle low (US$4,690 to <7,760); Low (<US$4,690) *P-value < 0.05.

## Discussion

### Social isolation and chronic disease prevalence

In this study, we found higher major chronic disease prevalence in elderly living alone than in elderly living with another adult. This finding was in line with the study by Kharicha et al., which showed an association of living alone with arthritis/rheumatism, glaucoma, and cataracts in older people [17]. The disadvantages of social isolation in personal health, especially mental health, have been reported in several studies as well [18].

Living arrangement is an important determinant of loneliness for older people [19]. In Asian countries under Confucian culture including South Korea, “*Hyo*” (filial piety), a concept respecting parents, was one of the most important values in society and children fulfilled these responsibilities by caring for their parents for their whole lives [20]. But, rapid industrialization in Korean societies has somewhat disrupted those cultures and has accelerated the separation of family members from each other [3]. Complementary actions for elderly welfare such as pensions and home care were introduced in the1980s under the Law of Elderly Welfare, but the social safety network provided by the government was not sufficient to keep pace with the rapid aging of society. The higher probability of having chronic disease in elderly living alone observed in this study could be explained by social isolation.

### Accessibility of prescription drugs depending on living arrangements

Elderly living alone spent less OOP money on prescription drugs than elderly living with other adults after adjusting for the number of major chronic diseases. We considered that the OOP prescription drug expenditure figures reflect the total prescription drug expenditure because the current national health insurance benefit scheme in Korea applies the universal co-insurance (30%) for total prescription drug expenditures including both NHI burden and patient burden. Therefore, this study result suggests that elderly living alone might potentially underuse prescription drugs even though they have a higher risk of chronic diseases.

There are possible relationships between living arrangements and prescription drug accessibility. Elderly living alone might self-neglect, even though they are more vulnerable to disease, resulting in less access to healthcare services. Self-neglect conditions prevented them from seeking care for themselves and led to refusal of appropriate basic services [7]. The higher burden of co-insurance for prescription drugs could be another reason for medication underuse among elderly living alone. A co-insurance program is one of the cost-sharing schemes currently being implemented in the NHI system; it attenuates financial safety for patients taking a number of medicines because OOP money usually increases with the number of diseases. As in previous studies reporting that OOP health care expenditures increased more sharply in patients with multiple morbidities [21], this study showed such findings as well (Table 3). Elderly living alone at a higher risk of chronic disease were expected to spend more OOP money for prescription drug, but they actually spent less money due to the personal burden of OOP money, we assumed. A 10% increase in cost-sharing was reported to reduce prescription drug use by 2-6% [22]. However, this negative impact by increased co-insurance was not equal for all people. Gruber et al. (2006) found that varied copayment did not influence the person at an average income level but had a negative effect on personal health for those at a low income level [23]. Gemille et al. (2008) reported that medication use in elderly was more sensitive to copayment level than that in non-elderly [14]. As shown in the example of Medicare Part D, drug coverage under a health plan is critical to relieve the prescription drug cost burden [24–26]. Thus, the impact of a cost-sharing scheme for the user, especially the elderly, should not be neglected in optimizing healthcare.

Changes in living arrangement patterns are unavoidable in modern society. Our concern is that potential medication underuse by elderly living alone with chronic diseases might lead to severe and complicated disease status, resulting in increased hospitalization and emergency visits. To overcome this problem, the healthcare system should continue to evolve in order to ensure social security. Since the long-term care insurance system for elderly was introduced in 2008 in South Korea, home nursing care service has expanded [27]. But, further strategies to overcome the social loneliness and isolation of the elderly, together with optimal healthcare use, are required simultaneously.

The results of this study need to be interpreted considering some limitations. First, this was a cross-sectional study and causal effect could not be identified. Second, the study population enrolled in this study was community dwelling elderly persons, and the results might not apply to all the elderly. And, this study included the results for living alone, living with spouse and living with adults who are not elderly, without elderly living with other elderly and with children which we excluded due to very small number of subject groups and in order to minimize a heterogeneity of the within group. Third, subjects enrolled in this study were asked to keep all prescription sheets and payment receipts during the study period to avoid recall bias. Information on OOP prescription drug expenditures were based on self-reported diary entries. A diary is considered a good tool for collecting diverse information reported on formal documents even though it has a possibility of underreporting. Fourth, the association between living arrangements and OOP medical (or prescription drug) expenditures was weakened after adjusting for social economic status (i.e. income level or education level). In other words, elderly living alone showed trends on lower OOP expenditures, but there was no statistical significance. We could assume that living arrangement possibly correlates with social economic status such as income or education in elderly groups. Finally, we did not adjust for severity of major chronic disease and medical expenditure differences by those severities due to lack of data. Despite, our study suggested the possibilities of potential medication underuse in elderly living alone regardless of higher prevalence of chronic disease.

Conclusively, living arrangements of the elderly affected chronic disease prevalence after adjusting for demographic, social, and economic factors. Elderly living alone had a higher risk of chronic disease compared to elderly living with adults, but spent less on OOP prescription drug expenditures. There was an association between income level and living arrangements for the elderly, and lower income for elderly living alone may influence the decrease in prescription drug expenditures. Optimal prescription drug use is important for elderly with chronic diseases to achieve good health outcomes and quality of life. Public health policies including pharmacy benefit plans should be supplemented for vulnerable sub-populations such as elderly living alone in order to optimize medical treatment for their chronic conditions. Further studies to explain the causal relationship between elderly living alone and underuse of healthcare are required.
